# NAD^+^ repletion attenuates obesity‐induced oocyte mitochondrial dysfunction and offspring metabolic abnormalities via a SIRT3‐dependent pathway

**DOI:** 10.1002/ctm2.628

**Published:** 2021-12-19

**Authors:** Qingling Yang, Yujiao Wang, Huan Wang, Hui Li, Jing Zhu, Luping Cong, Jianmin Xu, Wenhui Chen, Yuqing Jiang, Yingpu Sun

**Affiliations:** ^1^ Center for Reproductive Medicine The First Affiliated Hospital of Zhengzhou University Zhengzhou China; ^2^ Henan Key Laboratory of Reproduction and Genetics The First Affiliated Hospital of Zhengzhou University Zhengzhou China; ^3^ Henan Provincial Obstetrical and Gynecological Diseases (Reproductive Medicine) Clinical Research Center The First Affiliated Hospital of Zhengzhou University Zhengzhou China

Dear Editor,

Over‐nutrition in females causes subfertility and impairs offspring health, but the detail mechanisms and therapeutic strategies has not been well investigated.^1^, ^2^, ^3^ Nicotinamide adenine dinucleotide (NAD^+^) is an important cofactor that regulates mitochondrial functions.^4^, ^5^, ^6^ In this study, we revealed that obesity induced NAD^+^ decline in oocyte, while supplementation of NAD^+^ precursor, nicotinamide riboside (NR), in HFD‐fed mice alleviated subfertility and reduced metabolic dysfunction in offspring through a NAD^+^‐SIRT3‐dependent pathway.

An obese mouse model was established by feeding female mice a high‐fat diet (HFD) supplemented with or without NR (400 mg/kg/day) for 3 months beginning at the age of 4 weeks (Figure S1). The NAD^+^ levels in ovaries and MII oocytes from HFD mice decreased dramatically in comparison with controls. RT‐PCR results showed that the key rate‐limited NAD^+^ biosynthesis gene *Nampt* was marked decreased in the obese oocytes (Figure S4A). However, the NAD^+^ decrease was largely reduced by supplementation of NR (Figures 1A and 1B and S4A). Super‐ovulated oocyte number was decreased in HFD mice compared with control mice (Figure 1C). Conversely, NR supplementation led to significantly more oocytes in HFD mice (Figure 1C) in comparison with HFD mice. Furthermore, more morphological defects as shown by fragments were observed in HFD oocytes compared with control oocytes, whereas such defects were attenuated due to supplementation of NR (Figure 1D and E). In addition, HFD mice gave birth to less offspring compared with controls. However, the subfertility of HFD mice were attenuated by administration of NR increased (Figure 1F).

**FIGURE 1:**
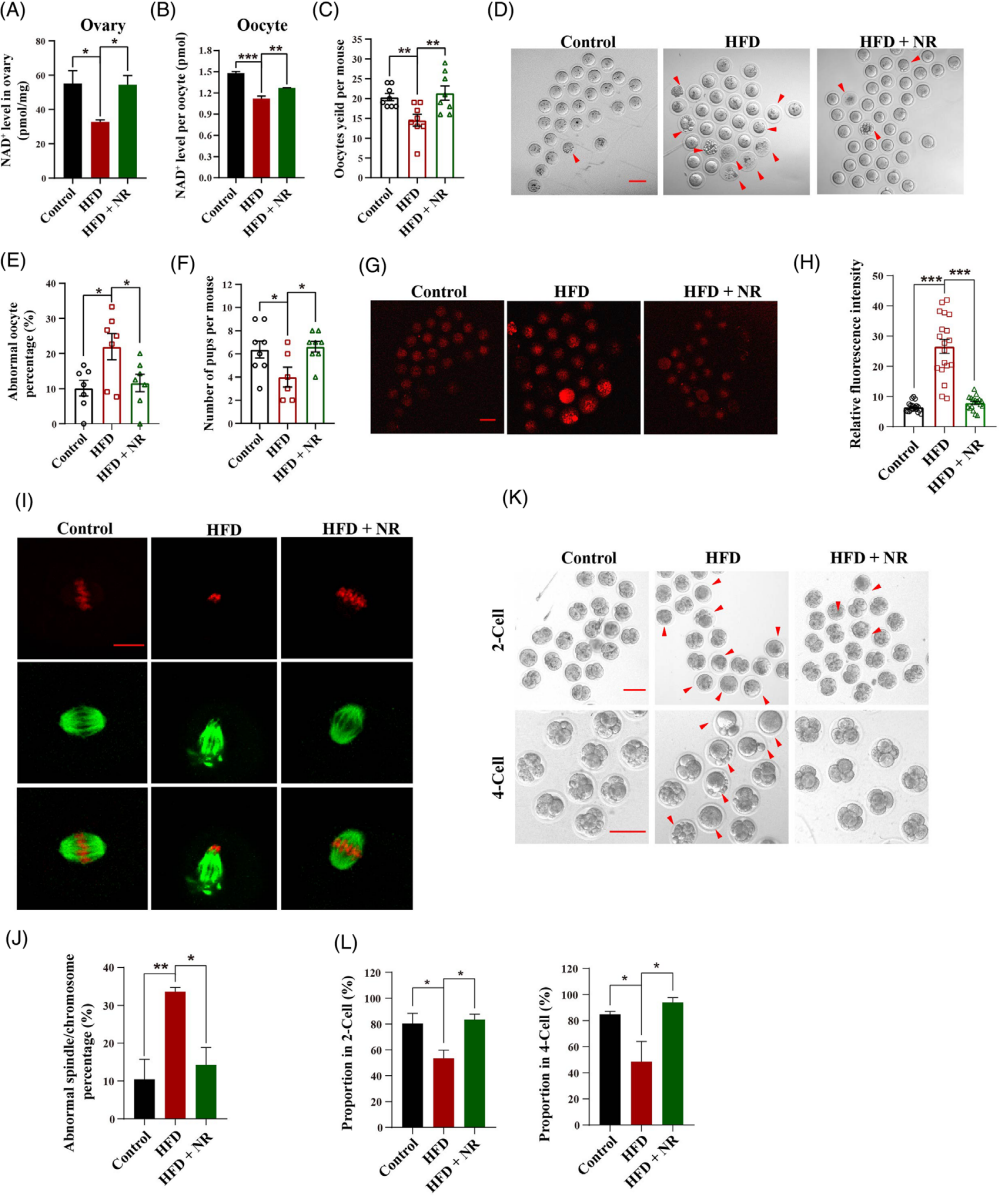
Supplementation of NR attenuates the obesity‐associated decline in oocyte quality and improves early embryonic competence by increasing the NAD^+^ level. (A) NAD^+^ levels in ovaries from Control, HFD and HFD supplemented with NR mice (*n* = 6 for Control, *n* = 4 for HFD and *n* = 6 for HFD + NR). (B) NAD^+^ levels in single oocytes from ND, HFD and HFD supplemented with NR mice (*n* = 6 for Control, *n* = 4 for HFD and *n* = 6 for HFD + NR). (C) Number of ovulated oocytes in mice of each group after gonadotropin induction of ovulation (*n* = 8 for each group). (D) Representative images of MII oocytes from Control, HFD and HFD mice with NR supplementation. Arrowheads indicate abnormal oocytes with cytoplasmic fragments. Bar = 10 μm. (E) Percentages of abnormal oocytes from Control, HFD and NR‐supplemented HFD mice (*n* = 7 mice for each group). (F) Average numbers of pups per mouse after mating with fertile male mice with a normal bodyweight (*n* = 8 for Control, *n* = 6 for HFD and *n* = 8 for HFD + NR). (G) Representative images of ROS staining with MitoSOX in oocytes from Control, HFD and HFD with NR supplementation mice. Bar = 100 μm. (H) Quantitative analysis of MitoSOX fluorescence intensity in oocytes from each group (*n* = 21 for Control, *n* = 19 for HFD and *n* = 20 for HFD + NR). (I) Representative morphologies of spindles and chromosome alignment in oocytes from Control, HFD and HFD mice with NR supplementation. An anti‐α‐tubulin antibody (green) was used to visualise spindles and propidium iodide (red) was used to visualise chromosomal DNA. Bar = 10 μm. (J) Percentages of Control, HFD and HFD + NR oocytes with spindle (disorganised or elongated)/chromosomal defects (misalignment) (*n* = 3 mice for each group). (K) Representative images of 2‐ and 4‐cell embryos from Control, HFD and HFD + NR mice. Red arrowheads show abnormal embryos with fragments. Bar = 100 μm. (L) Percentages of embryos at 2‐ and 4‐cell stages from Control, HFD and HFD + NR oocytes after in vitro fertilisation (*n* = 3 mice for each group). ^*^ *p *< .05, ^**^ *p *< .01, ^***^ *p *< .001

ROS content in MII oocytes was measured by using MitoSOX. The results showed that the ROS content was higher in HFD oocytes in comparison with controls, whereas NR supplementation significantly reduced ROS content in HFD oocytes (Figure 1G and H). MII oocytes from young mice displayed a typical barrel‐shaped spindle apparatus together with well‐organised chromosomes. Conversely, oocytes from HFD mice had a lower proportion of normal spindle morphology with well‐organised chromosomes, which was inhibited by NR supplementation (Figure 1I and J). Embryonic development was monitored after in vitro fertilisation, lower fertilisation rate and 4‐cell embryo formation rate in the HFD group were observed compared with the control group, whereas the decrease of embryonic development competence was alleviated after NR supplementation (Figure 1K and L).

To elucidate the effects of NR supplementation in the improvement of HFD quality, single oocyte transcriptome sequencing was performed and analyzed from control and HFD as well as HFD with NR mice. RNA‐seq data was confirmed by using RT‐PCR analysis of the randomly selected genes (Figure S2; Table S1 showed the primer sequences). Different gene expression trend was observed in HFD oocytes when comparing with controls, whereas NR treatment largely changed the obese oocyte transcriptome (Figure 2A and B). KEGG and GO analysis revealed that the differentially expressed genes were enriched in mitochondrial functions (Figure 2C–F). Consistent with the sequencing data, mitochondrial membrane potential was impaired in HFD oocytes as shown by a lower ratio of red to green fluorescence intensity (Figure 2G and H). Lower transcription of mitochondrial fusion and fission related genes was found in HFD oocytes (Figure 2I). In addition, ATP level together with OXPHOS related genes were all decreased in HFD oocytes in comparison with controls (Figure 2J and K). However, NR supplementation largely alleviated the changes in HFD oocytes. These observations further verified that NR supplementation improved mitochondrial functions in HFD oocytes.

**FIGURE 2:**
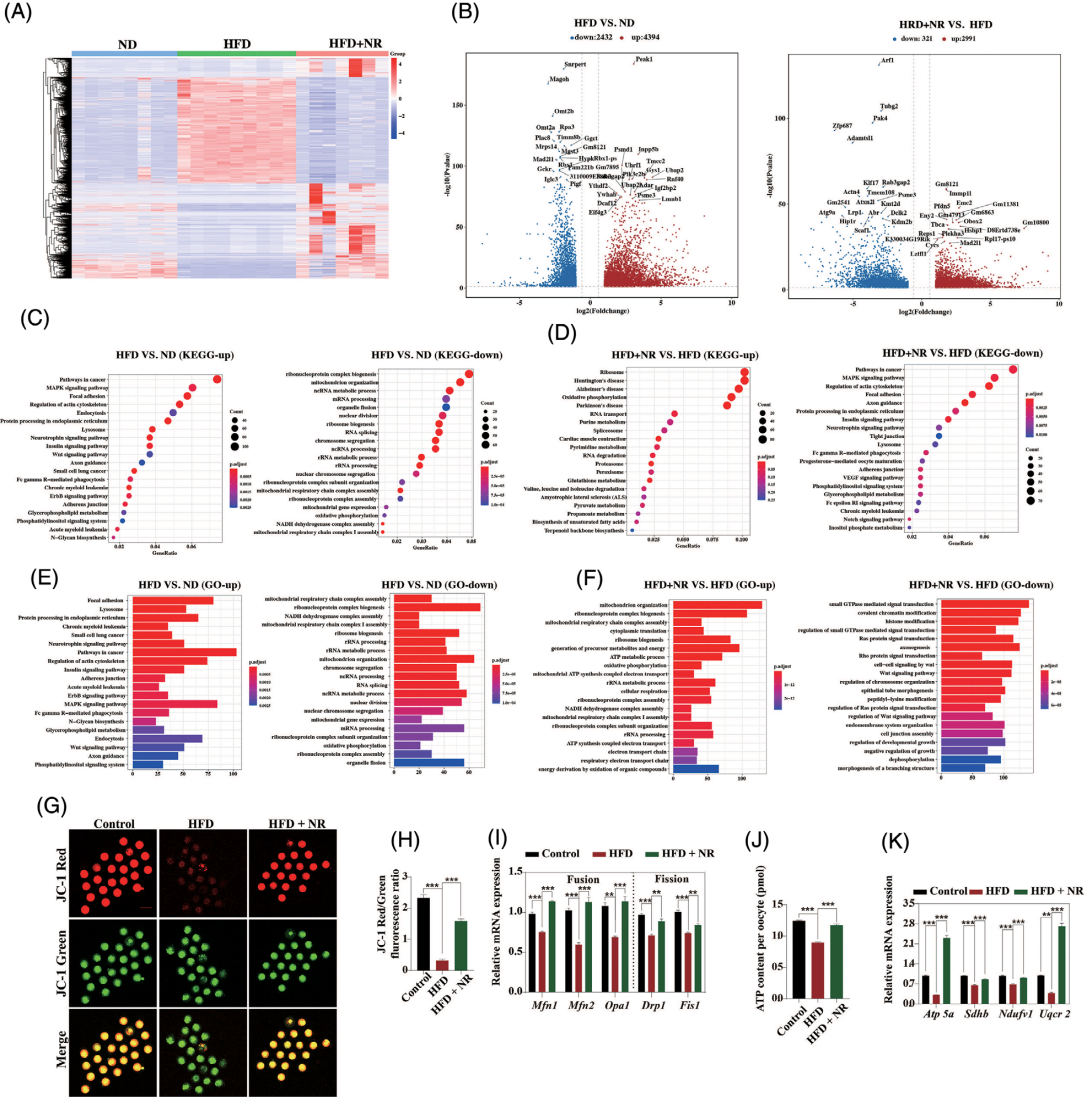
Supplementation of NR improves mitochondrial functions in HFD oocytes. (A) Whole transcriptome analysis in single oocyte from Control, HFD and HFD with supplementation of NR. Heatmap of mRNA expression profiles in oocytes from Control and HFD mice, as well as HFD mice with supplementation of NR. (B) Volcano plot showing differentially expressed genes in HFD oocytes compared with Control oocytes, as well as differentially expressed genes in HFD+NR oocytes compared with HFD oocytes. Blue: downregulated; red: upregulated. (C) KEGG enrichment analysis of upregulated and downregulated expressed genes between HFD oocytes and Control oocytes. (D) KEGG enrichment analysis of upregulated and downregulated expressed genes between HFD+NR oocytes and HFD oocytes. (E) Gene ontology (GO) analysis of upregulated and downregulated expressed genes between HFD oocytes and Control oocytes. (F) GO analysis of upregulated and downregulated expressed genes between HFD+NR oocytes and HFD oocytes. (G) Mitochondrial membrane potential was assessed in oocytes from Control, HFD and HFD mice with NR supplementation by JC‐1 staining. Green fluorescence shows inactive mitochondria, while red fluorescence shows active mitochondria in oocytes. The mitochondrial membrane potential was calculated by the ratio of red to green in each oocyte. Bar = 100 μm. (H) Histogram showing JC‐1 red‐to‐green fluorescence in oocytes from each group, which reflects mitochondrial activity (*n* = 23 oocytes for Control, *n* = 21 oocytes for HFD, *n* = 19 oocytes for HFD + NR). (I) Transcript levels of mitochondrial fusion (*mfn1*, *mfn2* and *opa1*) and fission (*Drp1* and *Fis1*) genes in ovary detected by real‐time RT‐PCR (*n* = 79 oocytes for Control, *n* = 84 oocytes for HFD and *n* = 47 oocytes for HFD + NR). (J) ATP levels in oocytes from Control, HFD and HFD mice with NR supplementation (*n* = 30 oocytes for ND, *n* = 40 oocytes for HFD and *n* = 53 oocytes for HFD + NR). (K) Transcription of genes related to OXPHOS in oocytes from each group (*n* = 79 oocytes for Control, *n* = 84 oocytes for HFD and *n* = 47 oocytes for HFD + NR). ^**^ *p *< .01, ^***^ *p *< .001

Accumulated evidence has validated that SIRT3, whose activity is activated by NAD^+^, is involved in the regulation of oxidative stress homeostasis in HFD oocytes.^7^, ^8^, ^9^ We found that HFD induced decreased transcription of *Sirt3* in oocytes but elevated by NR supplementation (Figure 3A), indicating that *Sirt3* may participate the protective effects of NR on HFD oocytes. To test this possibility, *Sirt3* knockout mice (genotyping was showed in Figure S3) were employed and treated as described above. We found that NR supplementation increased super‐ovulated oocyte numbers, but the effects was vanished, the abnormal oocyte morphology rate and ROS contents were still in higher levels in HFD treated *Sirt3* knockout mice (Figure 3B–F). The proportion of meiotic defects showed by abnormal spindle assembly/chromosome mis‐segregation was higher in HFD treated *Sirt3* knockout mice supplied with NR than wild type mice (Figire 3G and H). As expected, decreased mitochondrial membrane potential was observed in oocytes from HFD *Sirt3* knockout mice supplied with NR as compared with wild type mice (Figure 3I, E and J). These data together indicated that the effects of NR supplementation on improvement of mitochondrial function and HFD oocyte quality was largely dependent on *Sirt3*.

**FIGURE 3:**
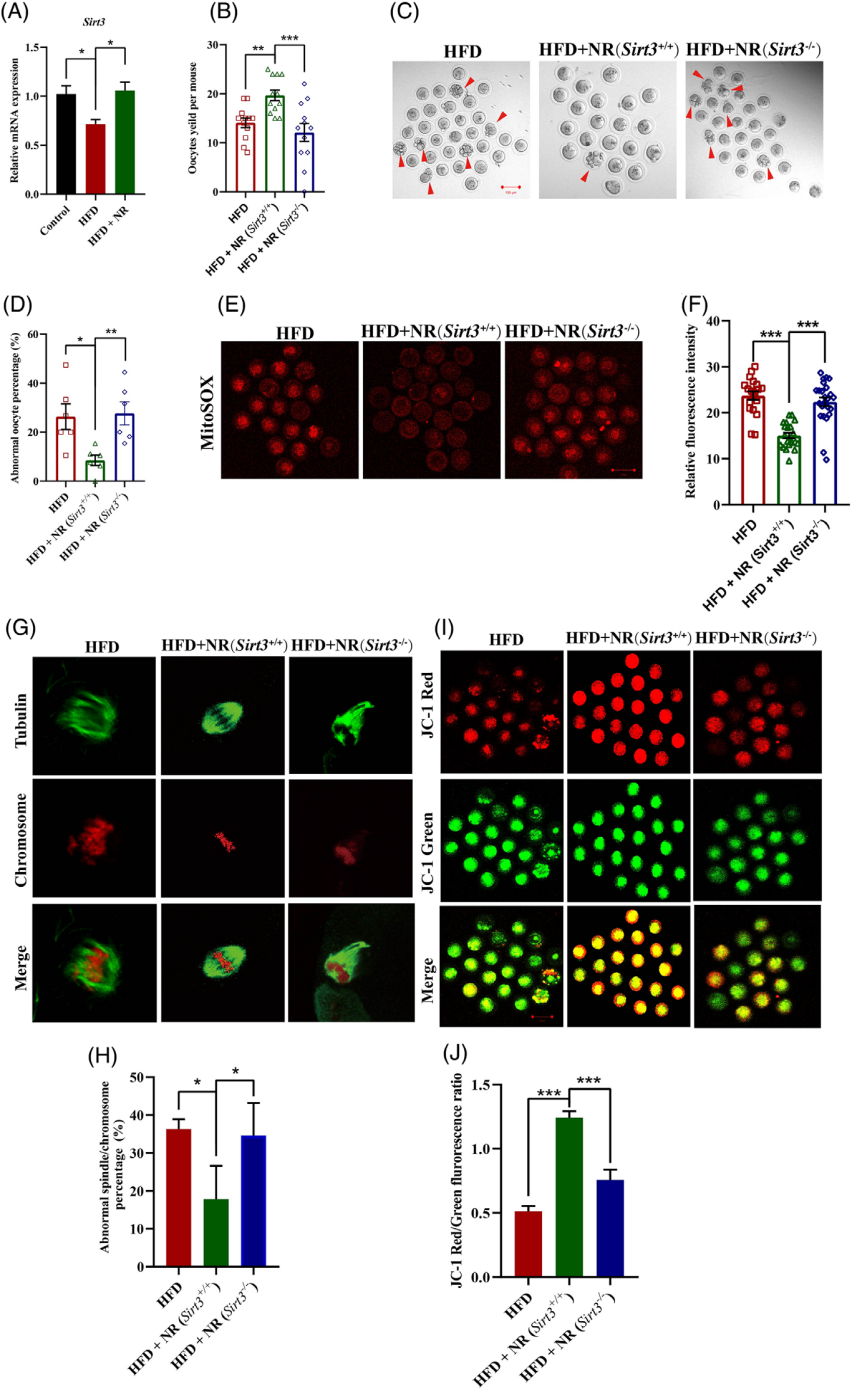
*Sirt3* depletion abolishes the improvement of HFD oocyte quality after NR supplementation. (A) Transcript levels of *Sirt3* in oocytes from HFD, HFD + NR (*Sirt3* ^−/−^) and HFD + NR (*Sirt3* ^+/+^) mice (*n* = 79 oocytes for Control, *n* = 84 oocytes for HFD and *n* = 47 oocytes for HFD + NR). (B) Number of ovulated oocytes in mice of each group after gonadotropin induction of ovulation (*n* = 12 for each group). (C) Representative images of oocyte morphologies in each group. Arrowheads indicate oocytes with fragments. (D) Percentages of abnormal oocytes with fragments from HFD, HFD + NR (*Sirt3* ^−/−^) and HFD + NR (*Sirt3* ^+/+^) mice. Bar = 100 μm. (E) Representative images of ROS stained with MitoSOX. Bar = 100 μm. (F) Quantification of ROS levels in oocytes from HFD, HFD + NR (*Sirt3* ^−/−^) and HFD + NR (*Sirt3* ^+/+^) mice [*n* = 19 oocytes for HFD, *n* = 21 oocytes for HFD+NR (*Sirt3^+/+^*) and *n* = 24 oocytes for HFD + NR (*Sirt3^−/−^*)]. (G) Representative morphology of spindles and chromosomal alignment in oocytes from each group. Spindles were visualised by staining with an anti‐α‐tubulin antibody (green) and chromosomal DNA was stained with PI (red). (H) Frequencies of oocytes with abnormal spindle morphology (disorganised or elongated) were determined in each group (*n* = 3 mice for each group) (I) Representative morphology of oocytes from each group stained with markers to reflect mitochondrial activity. (J) Ratio of red‐to‐green fluorescence as an indicator of the mitochondrial membrane potential in each group [*n* = 21 oocytes for HFD, *n* = 21 oocytes for HFD+NR (*Sirt3^+/+^*) and *n* = 17 oocytes for HFD + NR (*Sirt3^−/−^*)]. ^*^ *p *< .05, ^**^ *p *< .01, ^***^ *p *< .001

Because mitochondria are inherited maternally, we next investigated whether these beneficial effects of NR supplementation were transferred to offspring born to HFD mice. The results showed that glucose level was higher in offspring born to mice fed a HFD in comparison with those born to mice fed a control diet after injection of glucose. However, all of these changes were attenuated in the offspring born to mice fed a high‐fat diet supplemented with NR (Figure 4A and B). Mechanismly, NR supplementation increased expression of genes related to OXPHOS and NAD^+^ levels (Figure S4B) in offspring muscle born to HFD‐fed mice (Figure 4C and D), indicating that NR supplementation could alleviate obesity‐induced metabolic disturbances in offspring by improving mitochondrial functions.

**FIGURE 4:**
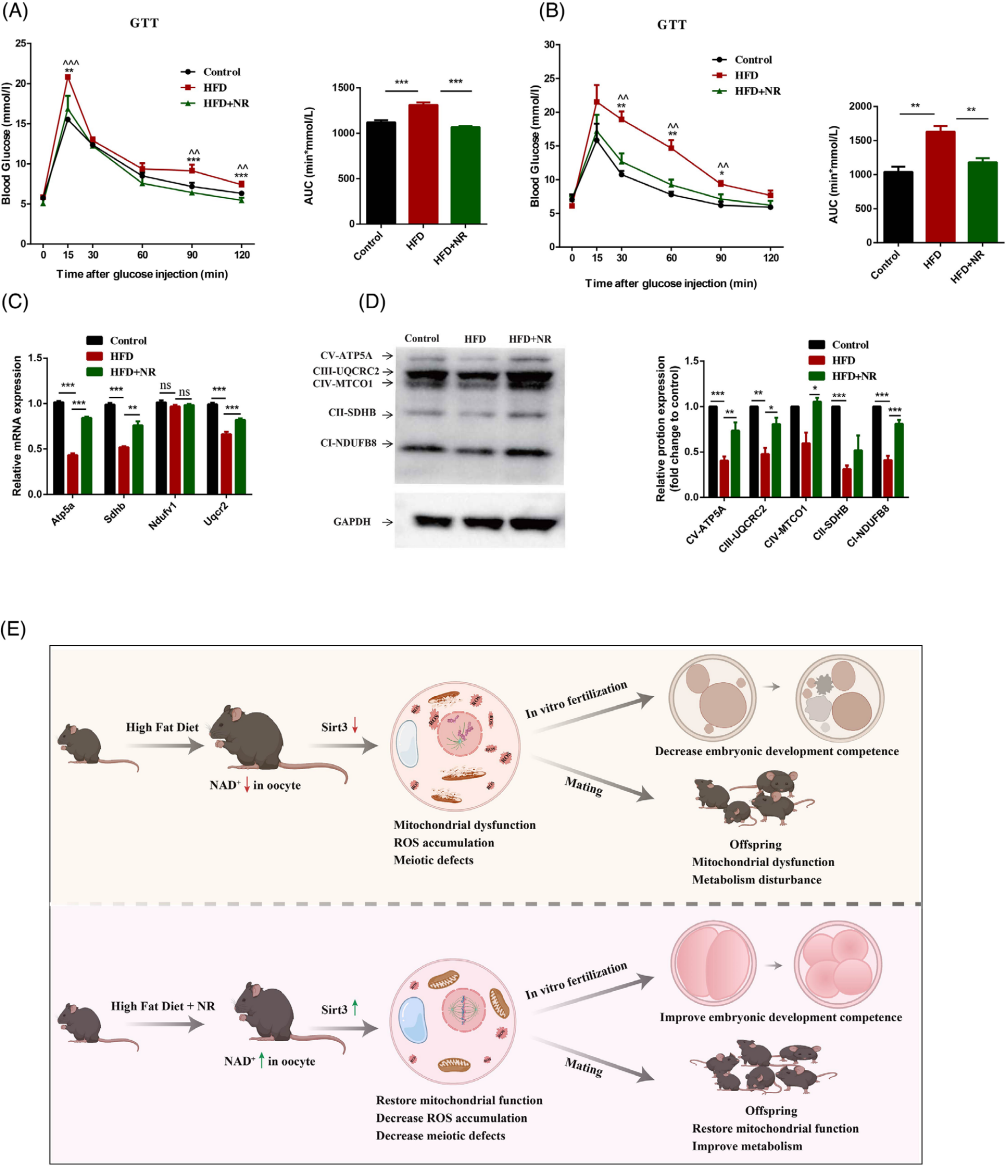
*Sirt3* mediated protective effects of NR supplementation on metabolism and mitochondrial function in offspring. GTT of 7‐ to 8‐week‐old female (A) and male (B) mice born to HFD‐fed dams, HFD+NR‐fed dams, or HFD + NR fed dams (*Sirt3* ^−/−^), the area under curve (AUC) were also calculated based on the glucose levels of each time point. ^*^Comparison between HFD and HFD+NR; ^Comparison of Control and HFD. (C) Transcription of genes related to OXPHOS in offspring's muscle from each group. (D) Immunoblotting for proteins related to electron transport chain complex content (CI, NDUFB8; CII, SDHB; CIII, UQCRC2; CIV, MTCO 1; CV, ATP5A). Relative expression of each protein was calculated as a ratio to GAPDH levels. (E) Diagram illustrating the beneficial effects of NR supplementation to ameroliate obesity‐induced oocyte dysfunction and improve offspring metabolism. Obesity decreases NAD^+^ levels in oocytes, which leads to mitochondrial dysfunction accompanied by ROS accumulation and meiotic defects, resulting in reduced oocyte quality and earlier embryonic developmental competence. NR supplementation restores mitochondrial functions in oocytes by restoring the NAD^+^ level and thus decreases ROS accumulation and meiotic defects via a Sirt3‐dependent pathway. Importantly, administration of NR attenuates the obesity‐induced decreases in early embryonic competence and the live birth rate as well as normalises the metabolism in offspring. ^*^ *p *< .05, ^**^ *p *< .01, ^***^ *p *< .001; ^^*p *< .01, ^^^*p *< .001

In summary, as shown in Figure 4E, HFD induced NAD^+^ deficiency in oocytes, which caused mitochondrial dysfunction, accumulation of ROS and abnormal spindle assembly, thereby impairing early embryonic development, and decreasing the live birth rate. However, increasing the NAD^+^ level by supplementation of NR to HFD mice improved mitochondrial functions in oocyte by via a Sirt3‐dependent pathway. Our data suggest that increasing NAD^+^ level is a potential therapeutic approach for treatment of obesity‐related ovarian infertility and metabolic disturbance in offspring.

## Supporting information


**FIGURE S1. Supplementation of NR attenuates HFD‐induced obesity and hyperglycaemia in mice**. (A) Workflow of establishing the obese mouse model through a high‐fat diet and supplementation of NR. (B) Representative images of Control, HFD and HFD with NR mice. (C) Bodyweights of female in each group were monitored during treatment from 4 to 12 weeks (*n* = 15 for each group). A GTT and ITT were performed for each group at 6 weeks (D and E) and 12 weeks (G and H), respectively. An area under curve (AUC) was calculated based on the glucose level at each time point. *Comparison between HFD and HFD+NR; ^Comparison of Control and HFD. *p < .05, **p < .01, ***p < .001; ^*p *< .05, ^^*p *< .01, ^^^*p *< .001

**FIGURE S2. Validation of RNA‐seq data by RT‐PCR**. (A) Randomly selected up‐ and downregulated genes in oocytes to verify the RNA‐seq data between in HFD and Control oocytes, (B) as well as between HFD and HFD + NR oocytes. ^*^ *p *< .05, ^**^ *p *< .01, ^***^ *p *< .001

**FIGURE S3. Generation of *Sirt3* ^−/−^ mice via the CRISPR/CAS9 system. ** (A)  Illustration of CRISPR/Cas9‐based targeting strategy to delete a 277‐bp sequence in *Sirt3* including exson 4. (B) PCR products of tail DNA from wild‐type mice and knockout mice. For genotyping of *Sirt3* ^−/−^ mice, PCR was performed on the DNA extracted from mouse tails, the *Sirt3* knockout mutant allele (837 bp) was assayed by primers 5′‐CAGTCAGTGACATCTTGGCTCTAC‐3′ (forward) and 5′‐ CAGCCCAGCCTTATGTTCCTTTAC‐3′ (reverse). The *Sirt3* wild type allele (611 bp) was assayed by primers 5′‐CAGTCAGTGACATCTTGGCTCTAC‐3′ (forward) and 5′‐CAAAGCAAATCTCAGTGTTGCAGC‐3′ (reverse). All animals were housed in a pathogen‐free environment in filter‐top cages

**FIGURE S4**. Transcription of genes related to NAD^+^‐Biosynthetic and consuming enzymes in oocytes from control and HFD mice (A) and NAD^+^ levels (B) in the offspring muscle from different group. ^*^ *p *< .05, ^**^ *p *< .01

Table S1
