# Supplementary figures and images for: NAD+ repletion attenuates obesity‐induced oocyte mitochondrial dysfunction and offspring metabolic abnormalities via a SIRT3‐dependent pathway
Source: Clin Transl Med. 2021 Dec 19;11(12):e628. doi: 10.1002/ctm2.628 (PMC8684772; doi:10.1002/ctm2.628)

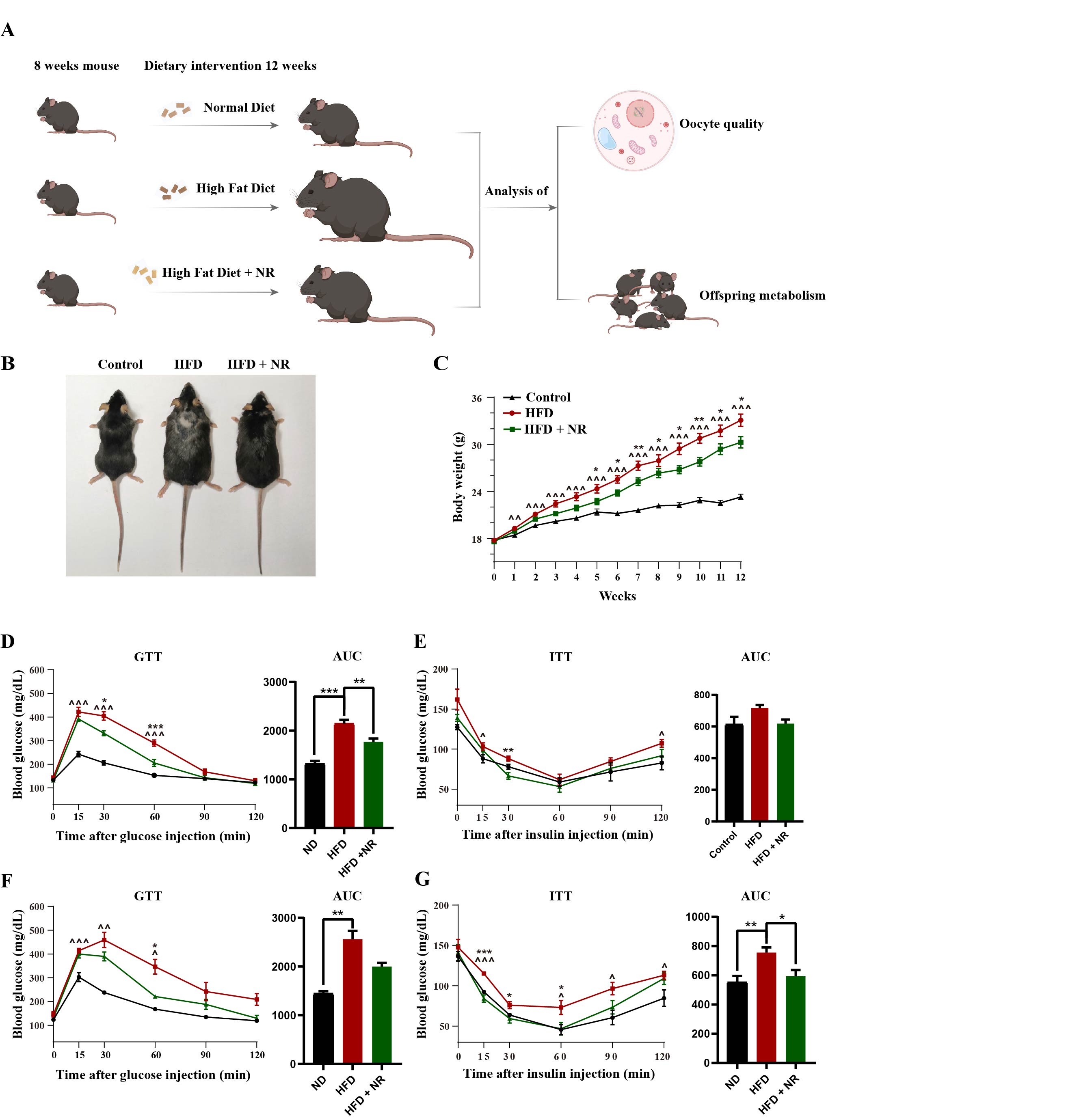

Supplement: Supplementary file 1 — FIGURE S1. Supplementation of NR attenuates HFD‐induced obesity and hyperglycaemia in mice. (A) Workflow of establishing the obese mouse model through a high‐fat diet and supplementation of NR. (B) Representative images of Control, HFD and HFD with NR mice. (C) Bodyweights of female in each group were monitored during treatment from 4 to 12 weeks (n = 15 for each group). A GTT and ITT were performed for each group at 6 weeks (D and E) and 12 weeks (G and H), respectively. An area under curve (AUC) was calculated based on the glucose level at each time point. *Comparison between HFD and HFD+NR; ^Comparison of Control and HFD. *p < .05, **p < .01, ***p < .001; ^p < .05, ^^p < .01, ^^^p < .001 [file CTM2-11-e628-s005.jpg]

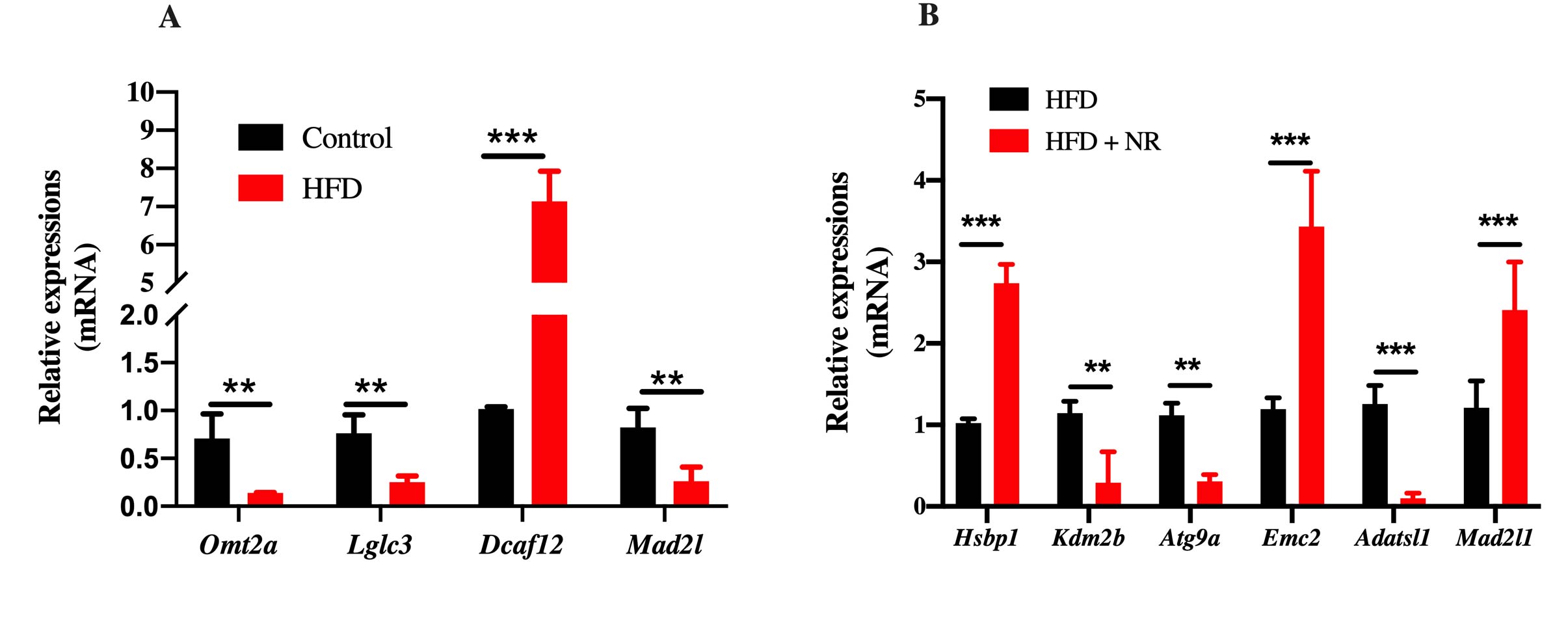

Supplement: Supplementary file 2 — FIGURE S2. Validation of RNA‐seq data by RT‐PCR. (A) Randomly selected up‐ and downregulated genes in oocytes to verify the RNA‐seq data between in HFD and Control oocytes, (B) as well as between HFD and HFD + NR oocytes. * p < .05, ** p < .01, *** p < .001 [file CTM2-11-e628-s001.jpg]

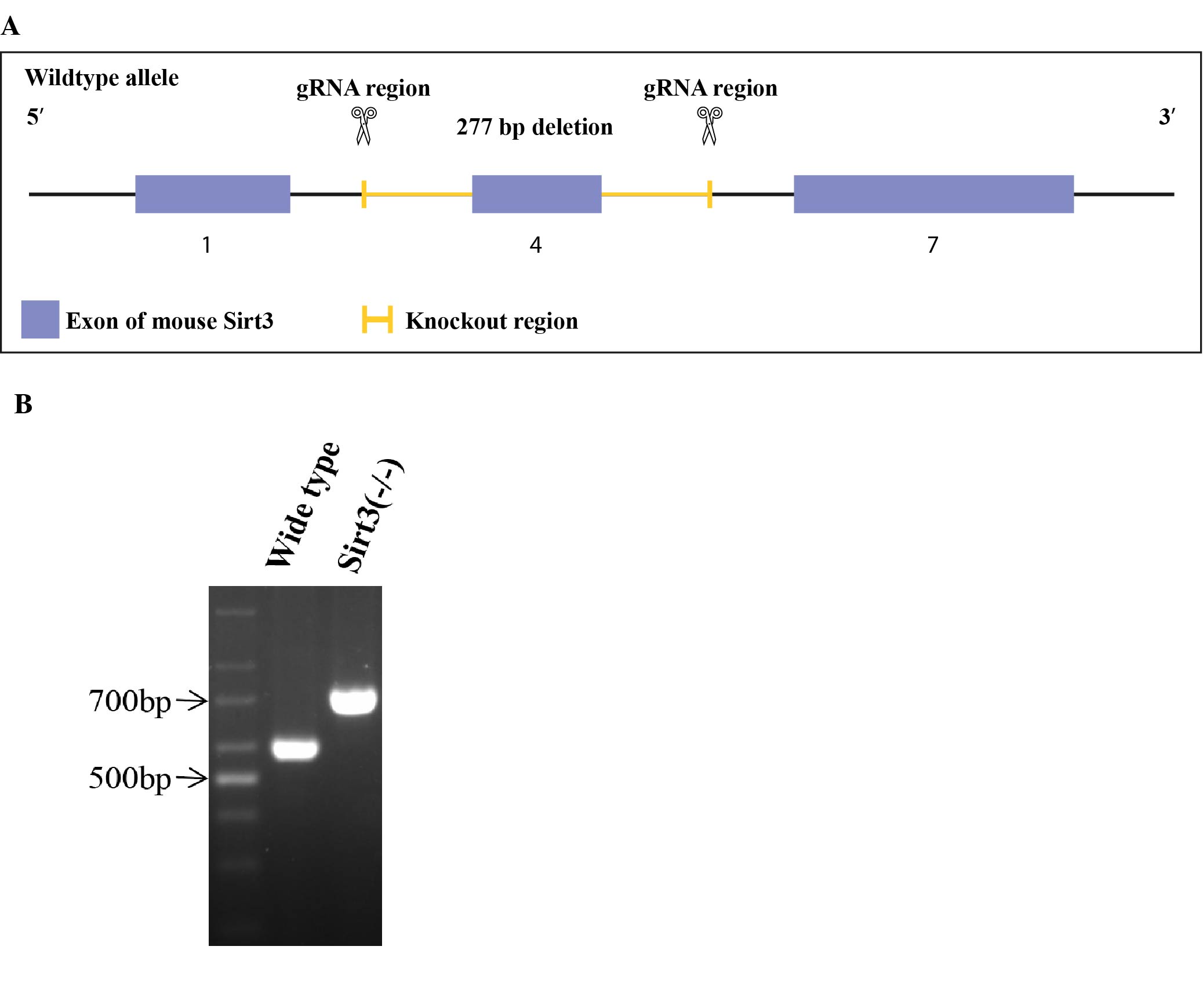

Supplement: Supplementary file 3 — FIGURE S3. Generation of Sirt3 −/− mice via the CRISPR/CAS9 system. (A) Illustration of CRISPR/Cas9‐based targeting strategy to delete a 277‐bp sequence in Sirt3 including exson 4. (B) PCR products of tail DNA from wild‐type mice and knockout mice. For genotyping of Sirt3 −/− mice, PCR was performed on the DNA extracted from mouse tails, the Sirt3 knockout mutant allele (837 bp) was assayed by primers 5′‐CAGTCAGTGACATCTTGGCTCTAC‐3′ (forward) and 5′‐ CAGCCCAGCCTTATGTTCCTTTAC‐3′ (reverse). The Sirt3 wild type allele (611 bp) was assayed by primers 5′‐CAGTCAGTGACATCTTGGCTCTAC‐3′ (forward) and 5′‐CAAAGCAAATCTCAGTGTTGCAGC‐3′ (reverse). All animals were housed in a pathogen‐free environment in filter‐top cages [file CTM2-11-e628-s002.jpg]

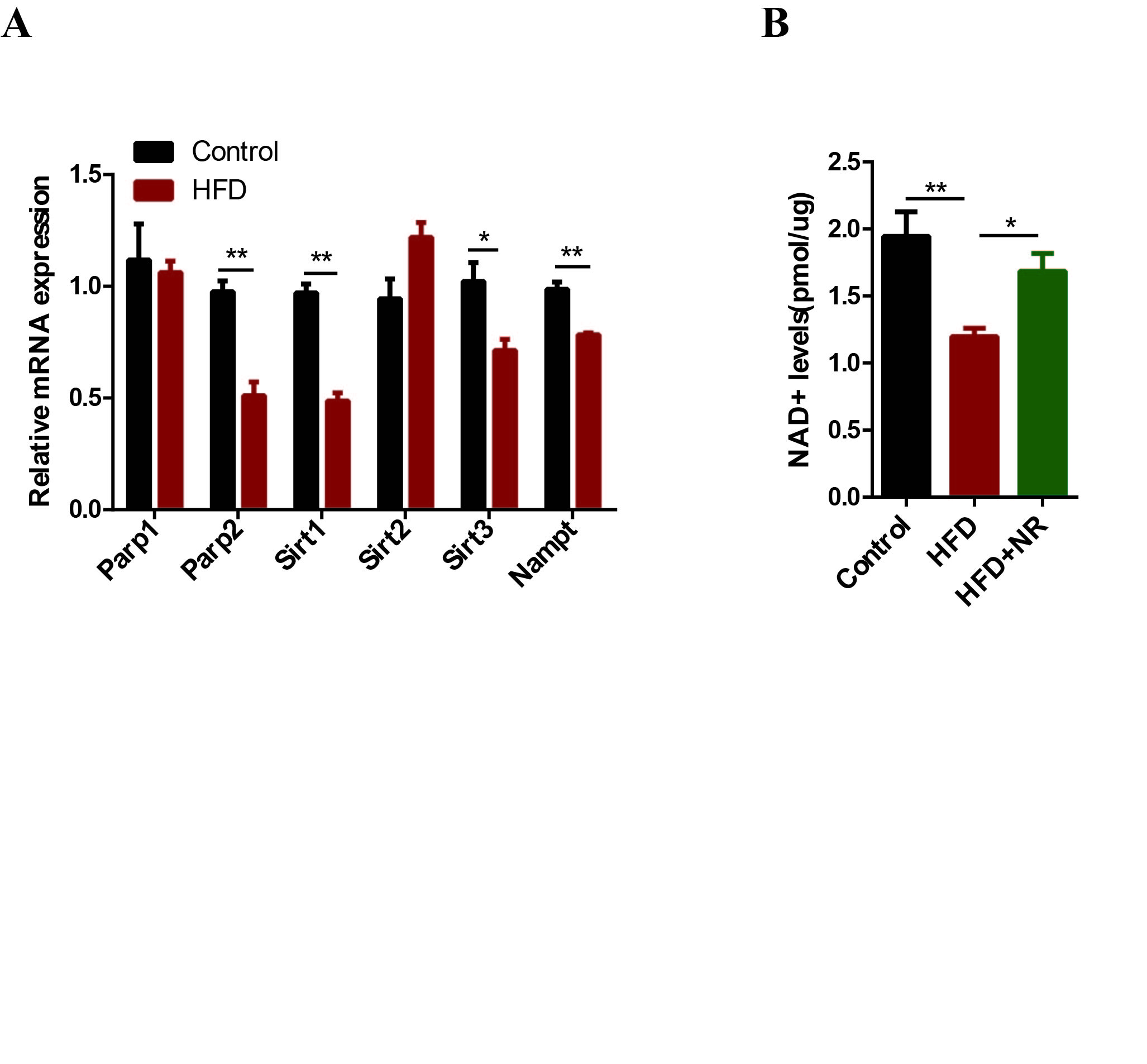

Supplement: Supplementary file 4 — FIGURE S4. Transcription of genes related to NAD+‐Biosynthetic and consuming enzymes in oocytes from control and HFD mice (A) and NAD+ levels (B) in the offspring muscle from different group. * p < .05, ** p < .01 [file CTM2-11-e628-s003.jpg]
