# Supplementary material for: NAD+ repletion attenuates obesity‐induced oocyte mitochondrial dysfunction and offspring metabolic abnormalities via a SIRT3‐dependent pathway
Source: Clin Transl Med. 2021 Dec 19;11(12):e628. doi: 10.1002/ctm2.628 (PMC8684772; doi:10.1002/ctm2.628)
Supplement: Supplementary file 5 — Table S1 [file CTM2-11-e628-s004.docx]

**Supplementary Table 1**

| Gene | Forward | Reverse |
| --- | --- | --- |
| *Mfn1* | CACTGCAATCTTCGGCCAGTTA | TTTCTGTAGCCCTGTATTTCCACCA |
| *Mfn2* | CTCCAAGTGTCCGCTCCTGAA | AGCTGTCCAGCTCCGTGGTA |
| *Opa1* | GCAGCATTAAGACATGAAATCGAAC | CCAGGGCCTTTGACATTTAGAGA |
| *Fis1* | TGGGCAACTACCGGCTCAA | TTATCAATCAGGCGTTCCAGCTC |
| *Drp1* | GTCCATGAGGAGATGCAGAGG | CACGGGCAACCTTTTACGA |
| *Atp5a* | AATCTCCATGCCTCTAACACTCGAC | GCAATACCATCACCAATGCTTAAC |
| *Sdhb* | ATCGACACGGACCTCAGCAA | GGTCCTCGATGGACTGCAGATA |
| *Ndufv1* | GCGGGTATCTGTGCGTTTCA | GCGCCCATACAGGTTGGTAAAG |
| *Uqcr2* | AACCCGTGGGATTGAAGCAG | CTGTGGTGACATTGAGCAGGAAC |
| *Iglc3* | CACTCCCACACTCACCATGTTTCC | CAGGCCACTGTCACACCACTTG |
| *Hsbp1* | GGAAGGATAGATGACATGAGCA | TTTCAGGGTCCAGTTCTTCTAC |
| *Emc2* | CATGCGATTTGAAGCTATGGAA | CTTCTTGGTCTCCAACAAACTG |
| *Kdm2b* | CTACGTAGCCAGTTATACCAGG | GAGCTTCTCTCTGTACGTACTC |
| *Atg9a* | CGTGAGCTGACAGAGTTGGACATC | GCAGAGGCAGGAGGGATTTGTTC |
| *Adamtsl1* | GACAGGTATGTGGAGACCAAAG | GAAGTCAGACAGGAACTCTTCA |
| *Dcaf12* | CTTGCCTTCTCCGCCTCATGTG | TTCCGCTTCCTGCTAACTGCTTTC |
| *Lmnb1* | TGAGGACTTGGAGTTTCGTAAA | TACTCATACTCAATCTGACGCC |
| *Inpp5b* | CTACTGCCACAAAACTCAACTC | CCAGACACAGACAGAAAGTAGT |
| *Omt2a* | GCCTGGAACCATAGTGTGCCTTG | ACCTTCTTCTAAATGCTGCCTTCTGTC |
| *Mad2l1* | AGAAAAGTCCCAGAAAGCCATA | GTGGTTGTAAATGAGCGTAGAC |
| *Gapdh* | AAATGGTGAAGGTCGGTGTGAAC | CAACAATCTCCACTTTGCCACTG |
